# Evaluating the Role of the Dorsolateral Prefrontal Cortex and Posterior Parietal Cortex in Memory-Guided Attention With Repetitive Transcranial Magnetic Stimulation

**DOI:** 10.3389/fnhum.2018.00236

**Published:** 2018-06-07

**Authors:** Min Wang, Ping Yang, Chaoyang Wan, Zhenlan Jin, Junjun Zhang, Ling Li

**Affiliations:** Key Laboratory for NeuroInformation of Ministry of Education, High-Field Magnetic Resonance Brain Imaging Key Laboratory of Sichuan Province, Center for Information in Medicine, School of Life Sciences and Technology, University of Electronic Science and Technology of China, Chengdu, China

**Keywords:** visual search, working memory, rDLPFC, rPPC, rTMS

## Abstract

The contents of working memory (WM) can affect the subsequent visual search performance, resulting in either beneficial or cost effects, when the visual search target is included in or spatially dissociated from the memorized contents, respectively. The right dorsolateral prefrontal cortex (rDLPFC) and the right posterior parietal cortex (rPPC) have been suggested to be associated with the congruence/incongruence effects of the WM content and the visual search target. Thus, in the present study, we investigated the role of the dorsolateral prefrontal cortex and the PPC in controlling the interaction between WM and attention during a visual search, using repetitive transcranial magnetic stimulation (rTMS). Subjects maintained a color in WM while performing a search task. The color cue contained the target (valid), the distractor (invalid) or did not reappear in the search display (neutral). Concurrent stimulation with the search onset showed that relative to rTMS over the vertex, rTMS over rPPC and rDLPFC further decreased the search reaction time, when the memory cue contained the search target. The results suggest that the rDLPFC and the rPPC are critical for controlling WM biases in human visual attention.

## Introduction

Working memory (WM) plays a crucial role in the control of visual selection. Behavioral and electrophysiological evidence show that WM biases the competition for selection in favor of objects that fit the WM content (Kumar et al., 2009; Hollingworth et al., 2013). Neuroimaging studies have implicated the involvement of the fronto-parietal cortical network in the control of attention (Buschman and Miller, 2007; Szczepanski et al., 2010) and in the maintenance of visual WM representations (Curtis and D’Esposito, 2003). However, it remains largely unknown how the fronto-parietal network controls the allocation of attention during a visual search, when a concurrent WM representation is maintained.

In the present study, we investigated the role of the dorsolateral prefrontal cortex (DLPFC) and the posterior parietal cortex (PPC) in controlling the interaction between WM and attention during a visual search. To this end we used transcranial magnetic stimulation (TMS), a technique that produces focal, transient and fully reversible disruptions in brain activity by delivering strong magnetic pulses to the cortex that pass through the skull and depolarize the underlying neurons of particular areas in the brain (Epstein et al., 2012; Balconi, 2013). This method has been proved to be a useful research approach to assess the functional and causal role of specific cortical areas in cognition (Miniussi and Rossini, 2011; Lage et al., 2016). TMS can be applied using single pulses or a train of pulses. The latter is referred to as repetitive TMS (rTMS). rTMS can induce after effects that outlast the stimulation period (Sandrini et al., 2011).

Dorsolateral prefrontal cortex is commonly associated with visual WM function (Postle et al., 2006; Preston et al., 2010), and in the control of memory-guided responses (Hamidi et al., 2010). To date, only few studies have measured the effects of DLPFC on visual search by using rTMS. For example, rTMS applied over the right DLPFC in healthy participants has been shown to trigger prolonged reaction times (RTs), in a conjunction search that requires cognitive coordination as compared with a pop-out search that is more of an automatic process (Kalla et al., 2009; Yan et al., 2016). It has been suggested that the DLPFC maintains a representation of a target and can thus induce top–down biases in order to guide the performance of the response to targets, while ignoring irrelevant distractors (Yan et al., 2016). These findings give further support to the proposition that prefrontal cortex is the source of top–down bias (Gazzaley et al., 2007; Rossi et al., 2007). In the present study we utilized a paradigm in which the WM component is significant, since the subjects need to memorize a primed color in each trial. Thus, it is likely that the DLPFC would have an important role in the memory-based biases during the visual selection process, which is why we chose this area as one of the target areas for stimulation.

The PPC, and specifically the right PPC (rPPC), has been implicated in WM (Todd and Marois, 2004; Xu and Chun, 2006), spatial attention (Corbetta et al., 2002) and visual search tasks (Lane et al., 2012, 2013). It has been shown to play a role in top–down selection of task-related targets (Hung et al., 2005). TMS of the PPC has been shown to delay the response times to targets during a visual conjunction but not a feature search (Fuggetta et al., 2006; Lane et al., 2011). Such a selective disruption of performance disappears if subjects are instructed to locate the target location, suggesting that the PPC has a specific role in coding the location of visual stimuli (Lane et al., 2011). Others have shown that TMS to the right PPC (but not the left PPC) facilitates visual search by reducing the RT cost of a salient singleton distractor, suggesting that rPPC-TMS reduces the attentional capture of irrelevant distractors even when these are salient (Hodsoll et al., 2009). Overall, the findings support the important role of the rPPC in top–down attentional control, a process which attempts to draw attention away from irrelevant distractors in order to focus on performing specific goals (Proulx and Egeth, 2006; Müller et al., 2009; Kiss and Eimer, 2011).

Given that the DLPFC and the PPC share many functional properties, it is of importance to compare the similarities and differences between these two areas in the attentional deployment, during a visual search when a concurrent WM representation is maintained. One way to compare the respective roles of these cortical areas is to manipulate the match relationship between the target location and the memory content. We used a paradigm based on a series of studies by Soto and Humphreys (2007) and Soto et al. (2007), where subjects maintained a color in WM and were asked to discriminate the orientation of a target. In a search display, the reappearance of a memorized color patch can either slow down the search for a target, if the color patch contains a distractor, or facilitate the search performance if the color patch contains a target. A previous fMRI study investigating human visual orientation found that a set of regions, which are predominantly located in the right cortical hemisphere, respond differently to valid versus invalid cued targets (Corbetta et al., 2002). In addition, the use of concurrent TMS-fMRI of the frontal and parietal cortices showed a right-hemisphere specialization for causal influences on the visual cortex (Ruff et al., 2009). Thus, in the present study we focused on the right PPC and the right DLPFC as the primary regions of interest.

**FIGURE 1:**
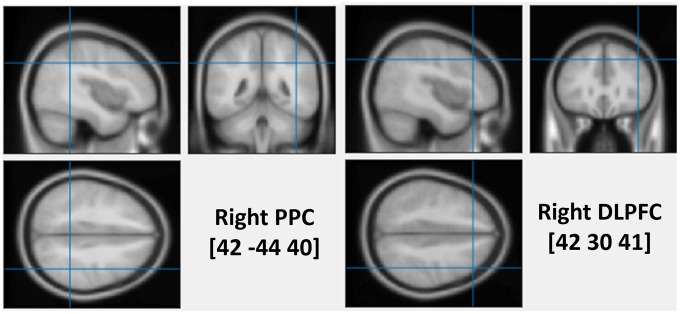
The average stimulated rPPC (MNI: 42, –44, 40) and rDLPFC (MNI: 42, 30, 41) sites. The position was verified using individual MRI scan co-registered to their head landmarks using BrainSight software.

To assess the causal role of the DLPFC and the PPC in determining the interaction between WM and attentional deployment, event-related rTMS was applied over the right DLPFC, the right PPC or the vertex, immediately after the onset of a visual search display. The vertex was selected as a control site for non-specific disruption of search performance due to concurrent discomfort, stimulation noise, and muscle twitches. Each event-related train of rTMS consisted of a 10 Hz, five-pulse delivery (500 ms in duration). Such 10 Hz stimulation protocol has been demonstrated to produce a suppressive effect on cognitive processing of the stimulated cortex, during visual search tasks (Lane et al., 2011; Muggleton et al., 2011; Taylor et al., 2011). The MNI coordinates that were used for the stimulation of the right DLPFC and the right PPC were (42 30 41, BA-46) and (42 -44 40, BA-40), respectively (see **Figure 1**). The coordinates of the rDLPFC corresponded to an area that showed an enhanced activity during the WM cue valid condition, and a decreased activity during the WM cue invalid condition (Soto et al., 2007), suggesting a modulatory role of the DLPFC on attentional control of WM cues and search targets. Thus, we hypothesized that the 10 Hz TMS stimulation of the rDLPFC would suppress its function, disturbing the WM biased effects on visual search (Kalla et al., 2009; Yan et al., 2016). This may eliminate both the WM facilitating effect in the valid condition and the cost effect in the invalid condition. A disruption of the WM effect at earlier stages of the visual search may result in a subsequent impaired WM performance. Alternatively, if the rDLPFC is responsible for a top–down attentional allocation toward the task-relevant search target, while ignoring the task-irrelevant WM content, we would expect an increased attentional capture of the color that is maintained in WM, resulting in a greater validity effect and improved WM performance.

The utilized coordinates for the rPPC correspond to an area that was suggested to suppress the distraction of irrelevant WM content during a visual search task (Soto et al., 2012b). Thus, we hypothesized that the 10 Hz TMS stimulation of the rPPC would suppress its function, and would result in an interference of the WM-match distractor during the search, leading to both faster search RTs in the valid condition and slower search RTs in the invalid condition. In this case, we would expect improved WM performance. Alternatively, if rPPC has a role in the top–down selection of task-relevant targets surrounded by distractors, stimulation of this area would result in increased search time (Fuggetta et al., 2006; Lane et al., 2011). Thus, we hypothesized that rPPC-TMS would impair the overall visual search performance, specifically when the WM content is spatially dissociated from the search target, thus drawing away attention during the invalid condition. In addition, we hypothesized that the reappearance of the memorized color in the search display would benefit the subsequent memory test, as subjects use the search display in order to ‘refresh’ the color representations thus facilitating the subsequent WM recognition (Soto et al., 2012a).

## Materials and Methods

### Subjects

Twenty-two right-handed students from University of Electronic Science and Technology of China (UESTC) were recruited for monetary compensation. All the subjects (11 females, mean age 21.68, range: 18–26) had normal color vision and had no history of neurological or psychiatric problems. The study was approved by the UESTC Ethics Board. Written informed consent was obtained from each subject prior to being tested. The methods were carried out in accordance with the approved guidelines and all experiments conformed to the Declaration of Helsinki.

### Stimuli and Task

All the stimuli used in this experiment were run through E-Prime 2.0 (Psychology Software Tools, Pittsburgh, PA, United States) on a computer monitor (41 cm × 23 cm) at 1366 × 768 pixels’ resolution with a refresh rate of 60 Hz. The stimuli consisted of colored disks (radius: 1.5°), embedded by a letter ‘T’ or ‘L’ (0.8° × 1.2°) at one of four possible orientations (upright, inverted, rotated 90° clockwise, or rotated 90° counterclockwise). The stimuli appeared against a black background with a central, white fixation cross subtending 0.38°.

**FIGURE 2:**
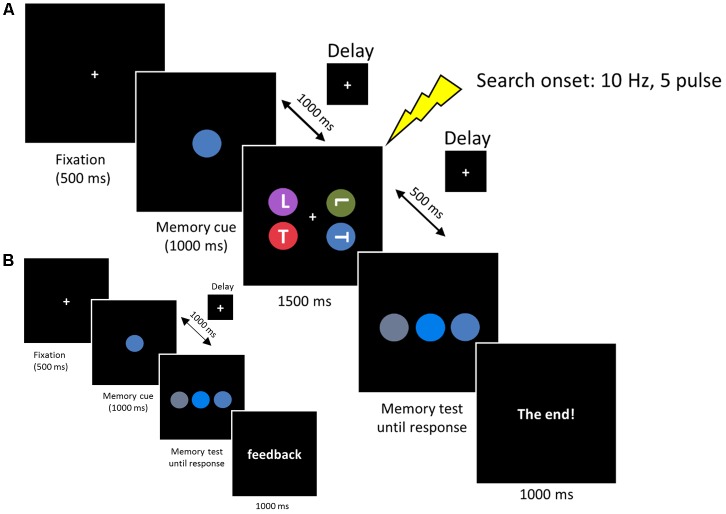
**(A)** Each trial began with a 500 ms fixation, then the memory cue was presented for 1000 ms. Subjects were asked to memorize the cue for a subsequent memory test. In the retention period, a visual search display was presented for 1500 ms and subjects reported the orientation of the target ‘T’ (an upright ‘T’ or an inverted ‘T’). The memorized cue indicated the target location (valid), the distractor location (invalid) or did not reappear (neutral). In the memory test display, subjects reported the original memorized color within the same color category. **(B)** Memory test practice prior to formal experiment. The procedure was the same as the experimental session, except that the search task was removed and a feedback was added at the end of each trial, informing subjects whether they made a correct response.

Each trial started with a central fixation for 500 ms, followed by the WM cue disk that was presented for 1000 ms. Subjects were required to remember the color accurately. After a delay of 1000 ms, the search arrays were presented for 1500 ms. Subjects were instructed to look for two possible targets (an upright ‘T’ or inverted ‘T’), one of which was presented on each trial. Subjects reported the orientation of the target by pressing, as quickly as possible, the key ‘1’ or ‘2’ on the numerical keyboard, with the index or middle finger of their right hand, respectively. The target appeared with equal probability at one of the four lateral positions (11.6° × 11.6°) and the other three disks always contained one ‘T’ and two ‘L’s to ensure that the letter ‘T’ and ‘L’ occurred equally. After a 500 ms delay, the memory test display was presented until a button response was recorded. Subjects were required to choose the initial memory cue from three stimuli by pressing key ‘1,’ ‘2,’ or ‘3’ on the numerical keyboard, with the right index, middle, or ring finger, respectively. After the offset of the memory test display a final “end” screen was displayed for 1000 ms (‘The end!’). Successive trials began immediately after this final display (see **Figure 2A**).

Colors were chosen from five main colors (red, blue, green, purple, or orange). We fixed the hue and value (brightness) of each color and varied the chroma to produce three different colors based on Munsell’s (1929) color system. The different color combinations used in this experiment are listed in **Table 1**. Note that the colors used in the memory test display were chosen from the same color category, thus minimizing the role of verbal encoding. In the search display, no two colors were selected from the same color category. Three conditions were included in the search display. In the first condition (valid condition), the memory cue contained the target letter. In the second condition (invalid condition), the memory cue contained a distracter letter. In the third condition (neutral condition), the memory cue did not reappear in the search display. All the subjects received a memory test practice before the formal experiment. The procedure for the practice test was the same as that described above, except that the search task was removed and a feedback was added at the end of each trial, informing subjects whether they made the correct response (see **Figure 2B**). This familiarized the subjects with the color stimuli and ensured they could discriminate the colors within the same category.

**Table 1:** The colors used in the experiment.

Munsell (as chosen)				RGB (as measured)		
Hue	Value	Chroma		R	G	B
			Red			
5R	5	6		168	105	101
5R	5	10		194	90	87
5R	5	16		228	56	69
			Purple			
5P	5	6		132	116	142
5P	5	10		149	104	172
5P	5	16		167	88	201
			Orange			
5YR	5	4		151	114	89
5YR	5	8		172	106	52
5YR	5	14		190	97	0
			Blue			
5PB	5	4		108	122	148
5PB	5	10		74	123	190
5PB	5	16		0	124	235
			Green			
5GY	5	2		117	124	102
5GY	5	6		109	129	59
5GY	5	12		98	133	0


*The Munsell values (R = red; P = purple; Y = yellow; B = blue G = green)were converted to red, green, and blue (RGB) values by a computer program called Munsell Conversion (Gretagmacbeth, 2004) (Available from www.gretagmacbeth.com).*

Three rTMS sessions were included: rDLPFC, rPPC, and vertex TMS. Each had a session consisted of three blocks of 30 trials, which contained 10 valid trials, 10 invalid trials, and 10 neutral trials. The trials were randomly intermixed within a block and the order of the rTMS sessions was random and counterbalanced across all the subjects. At least 30 min of rest was required between the sessions, to allow for the rTMS effect to wash out. Before each rTMS session, a no-TMS block was performed to prepare subjects for the following test. Practice blocks were performed before starting the actual experiment, and the subjects who could not discriminate between the colors or who were unable to maintain eye fixation were excluded from the study. Subjects were not aware of the valid, invalid and neutral conditions before the experiment. During the experiment, the subjects’ head was maintained in a fixed position using a chinrest. Eye movements were monitored with a video camera mounted behind the computer. A visual inspection determined that none of the trials had to be eliminated due to excessive eye movements.

### TMS and Stimulation Sites

Repetitive TMS was applied using a Magstim super rapid magnetic stimulator and an air-cooled figure-of-eight coil having an outer winding diameter of 70 mm (Magstim Company Limited, Whiteland, United Kingdom). rTMS was delivered at 10 Hz in trains of five pulses, concurrent with the search onset, inducing cortical inhibition at the site of stimulation (Lane et al., 2011; Muggleton et al., 2011; Taylor et al., 2011). The train of rTMS was 500-ms long (five pulses at 10 Hz). The average inter-train interval was approximately 7 s depending on how fast the subjects responded during the memory test. The vertex was selected as a control site for non-specific disruption of search performance due to concurrent discomfort, tactile sensations over the scalp, stimulation noise and muscle twitches. Since the motor cortex excitability does not provide reliable TMS thresholds in other cortical areas (Stewart et al., 2001; Muggleton et al., 2011; Kiyonaga et al., 2014), we did not use it as a reference for stimulus intensity. Stimulation intensity was delivered at a fixed intensity of 45% of the maximum stimulator output.

High-resolution anatomical T1-weighted magnetic resonance imaging (MRI) was acquired with a 3.0 T GE Sigma scanner. The scanner parameters were set as TR = 2000 ms, TE = 30 ms, flip angle = 90°, FOV = 240 mm, 70 slices and 1 mm thickness. rTMS stimulation sites were localized in individual participants using a frameless stereotaxy system (BrainSight Frameless, Rogue Research, Montreal, QC, Canada). Landmarks on the participants’ head were co-registered to individual MRI anatomical scans using this system. The rPPC site in MNI space (x, y, z) is 42, -44, 40. The rDLPFC site in MNI space (x, y, z) is 42, 30, 41. These coordinates were then used as stimulation targets and the TMS coil was placed on the corresponding location over the participants’ scalp. BrainSight was used to track the position of the TMS coil throughout the stimulation period, ensuring that it remained on the target location. The TMS stimulation site of the vertex was localized as a midpoint between the inion and the nasion and equidistant from the left and right ear.

### Data Analysis

The mean accuracy for the search task and mean RTs for the correct search trials were evaluated using the correct WM trials across the subjects. In addition, the mean accuracy and mean RTs of the correct WM trials were evaluated for the WM probe performance. In order to incorporate both RT and accuracy in a single measure, statistical analyses were also performed on the adjusted RTs for the search task (adjRTs = RTs/proportion of correct response) (Bardi et al., 2013; Bona et al., 2014). A repeated measure analysis of variance (ANOVA) was performed to compare search performance and memory performance, with condition (valid, invalid, and neutral) and TMS site (rDLPFC, rPPC, and vertex) as within-subject factors. *Post hoc t*-tests with Bonferroni correction for multiple comparisons were applied when necessary. Mean values ± standard error of the mean (SEM) are reported for the behavioral results. All the statistical analysis was performed using SPSS Statistics Release 19 (IBM, Somers, NY, United States).

## Results

**FIGURE 3:**
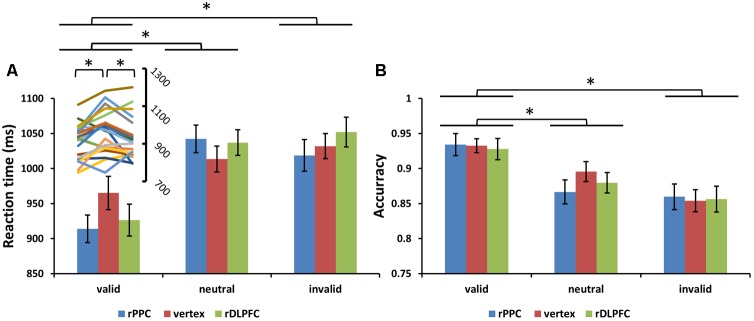
**(A)** Mean search reaction times (RTs) and **(B)** mean search accuracies are shown for the 22 subjects for all validity conditions across different stimulation sites (error bars show SEM). The line chart above the valid condition represent the individual RT variability across TMS stimulation sites. Asterisks mark significant *post hoc t*-test (^∗^*p <* 0.05).

Subjects correctly reported the location of the memory cue with a mean accuracy of 83.25% (*SD* = 6.01%). A two-way ANOVA comparing search performance with condition (valid, invalid, and neutral) and TMS site (rDLPFC, rPPC, and vertex) as factors revealed a significant main effect for condition [search ACC: *F*(2,42) = 14.170, *p* < 0.001, and partial eta square $\eta_{p}^{2}$ = 0.586; search RTs: *F*(2,42) = 26.087, *p* < 0.001, and $\eta_{p}^{2}$ = 0.723]. *Post hoc t*-tests using Bonferroni correction for the three possible comparisons across the three validity conditions revealed greater search ACC and faster search RTs in the valid condition compared to the neutral and invalid conditions [valid ACC and neutral ACC: *t*(21) = 4.250, *p* < 0.01; valid ACC and invalid ACC: *t*(21) = 5.357, *p* < 0.001; invalid ACC and neutral ACC: *t*(21) = 1.846, *p* > 0.1; valid RTs and neutral RTs: *t*(21) = 6.770, *p* < 0.001; valid RTs and invalid RTs: *t*(21) = 4.554, *p* = 0.001; invalid RTs and neutral RTs: *t*(21) = 0.292, *p* > 0.1]. No significant main effects for the TMS site were observed both for the search ACC and the search RT (all *p* > 0.1). No significant interaction between condition and TMS site was observed for the search ACC (*p* > 0.1). While, a significant condition × TMS site interaction was observed for the search RTs [*F*(4,84) = 3.805, *p* < 0.05, and $\eta_{p}^{2}$ = 0.458], showing that the TMS effect was only detected in the valid condition [*F*(2,42) = 6.972, *p* < 0.01, and $\eta_{p}^{2}$ = 0.411] but not in neutral and invalid conditions [all *p* > 0.1]. *Post hoc t*-tests, using Bonferroni correction for the three possible comparisons across the three TMS conditions, revealed faster search RTs in the rDLPFC-TMS compared to the vertex-TMS [*t*(21) = 2.816, *p* < 0.05], and faster search RTs in the rPPC-TMS compared to the vertex-TMS [*t*(21) = 3.424, *p* < 0.01] for the valid trials. **Figure 3** displays the search performance for correct WM trials for each level of the cue validity (valid, neutral, and invalid), at different rTMS stimulation sites (rDLPFC, rPPC, and vertex), pooled across the right and left visual fields.

**FIGURE 4:**
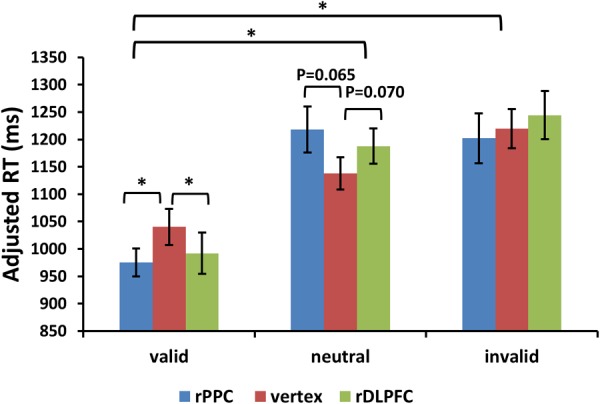
Adjusted search reaction times (adjRTs = RTs/proportion of correct response) are shown for the 22 subjects for all validity conditions across different stimulation sites (error bars show SEM). Asterisks mark significant *post hoc t*-test (^∗^*p <* 0.05).

For the adjusted search RTs, the same two-way ANOVA, using validity and TMS sites as factors, showed a significant main effect of validity [*F*(2,42) = 23.504, *p* < 0.001, and partial eta square $\eta_{p}^{2}$ = 0.702]. *Post hoc t*-tests revealed improved search performance in the valid condition compared to the neutral and invalid conditions [*post hoc t*-tests using Bonferroni correction for the three possible comparisons across the three validity conditions: valid and neutral: *t*(21) = 7.025, *p* < 0.001; valid and invalid: *t*(21) = 5.392, *p* < 0.001; invalid and neutral: *t*(21) = 1.497, *p* > 0.1]. No significant main effects for the TMS site were observed (*p* > 0.1). There was a significant validity × TMS site interaction [*F*(4,84) = 3.455, *p* < 0.05, and $\eta_{p}^{2}$ = 0.434], showing that the TMS effect was only detected in the valid condition [*F*(2,42) = 3.297, *p* < 0.05, and $\eta_{p}^{2}$ = 0.305] but not in neutral and invalid conditions [all *p* > 0.1]. *Post hoc t*-tests showed faster search adjRTs in the rPPC-TMS compared to the vertex-TMS [*t*(21) = 2.570, *p* < 0.05], and faster search adjRTs in the rDLPFC-TMS compared to the vertex-TMS [*t*(21) = 2.121, *p* < 0.05] for the valid trials. In addition, there was a marginally significant trend for longer search adjRTs for the rPPC-TMS compared to the vertex-TMS [*t*(21) = 1.944, *p* = 0.065], and a marginally significant trend for longer search adjRTs for the rDLPFC-TMS compared to the vertex-TMS [*t*(21) = 1.911, *p* = 0.070] for the neutral trials. **Figure 4** displays the adjusted search RTs for correct WM trials for each level of the cue validity (valid, neutral, and invalid), at different rTMS stimulation sites (rDLPFC, rPPC, and vertex), pooled across the right and left visual fields.

**FIGURE 5:**
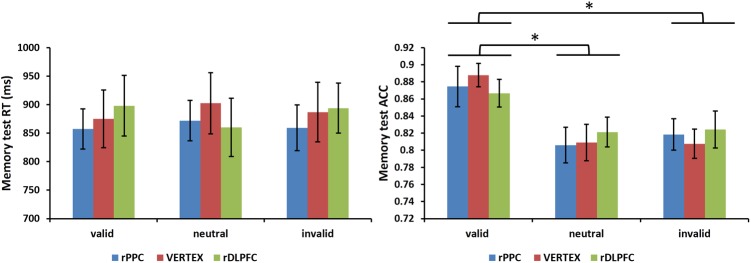
Mean memory test RTs of correct WM trials and mean memory test accuracies are shown for the 22 subjects for all validity conditions across different stimulation sites (error bars show SEM). Asterisks mark significant *post hoc t*-test (^∗^*p <* 0.05).

Furthermore, we assessed the effects of the cue validity and TMS stimulation site on the performance of a subsequent memory test. A 3 condition (valid, invalid, and neutral) × 3 TMS site (rDLPFC, rPPC, and vertex) ANOVA, comparing the mean RTs of correct WM trials, showed no significant main effects of validity, and TMS site, nor a significant interaction (all *p* > 0.1). For the memory test accuracy, the same two-way ANOVA showed no main effect for TMS site nor a significant interaction (all *p* > 0.1), but there was a significant main effect for cue validity [*F*(2,42) = 6.528, *p* < 0.01, and $\eta_{p}^{2}$ = 0.612]. *Post hoc t*-tests, using Bonferroni correction for the three possible comparisons across the three validity conditions, revealed greater memory test ACC in the valid condition compared to the neutral and invalid conditions [valid ACC and neutral ACC: *t*(21) = 3.727, *p* < 0.01; valid ACC and invalid ACC: *t*(21) = 3.400, *p* < 0.01]. No significant differences were found between the invalid condition and the neutral condition (*p* > 0.1). **Figure 5** displays the memory test performance.

## Discussion

The current study sought to identify the role of the DLPFC and the PPC in modulating the effect of internal WM content on attention, using TMS. The critical findings were that compared to vertex-TMS, rDLPFC-TMS, and rPPC-TMS induced faster RTs in the valid condition.

The behavioral results suggest that the memorized color may bias the recruitment of visual attention toward the target location, regardless of stimulation site, as indicated by greater search accuracies and faster search RTs during the valid condition relative to the neutral and invalid conditions. This is consistent with several findings in the literature regarding memory-based attentional capture (Soto et al., 2006b; Kim and Cho, 2016). In the current study, the activation of the search target template and the memorized template accumulated during the visual search and together contributed to the subjects’ response. Thus, subjects became more responsive to this conjoint activation inducing faster search response times in the valid condition. However, no significant costs of invalid trials were observed (no difference between neutral and invalid trials). This may be due to the utilized longer search presentation duration (1500 ms) compared to other studies that used shorter target displays (<200 ms) (Mazza et al., 2011; Soto et al., 2012c), thus increasing the perceptual difficulty of the search task. When the task is more difficult, the effect of the WM-match distractor may be enhanced, leading to invalid cost effects. In comparison, in the present study, the longer duration of the search display may have reduced uncertainty, thus decreasing the cost effects of the WM-match distractors. Another possibility may be linked to the fact that these studies used different colored shapes, and the combination of the two features of color and shape may have attracted more attention (Soto et al., 2005). In our study, the color-match distractor may have not been as conspicuous as the other non-primed colors during the invalid trials, especially given that the display size is four. On the other hand in the valid condition, the target information may have been sufficient to guide attention and thus the effect is enhanced when the target’s location matches that of the WM prime. Other similar studies, show that WM-match distractors during a visual search have either cost effects (Soto et al., 2007, 2011, 2012a), benefit effects (Soto et al., 2006a), or both (Mazza et al., 2011). These inconsistencies suggest that WM effects do not always appear at the same time using this specific paradigm.

In addition, a significant interaction between condition and TMS site was observed for the search RTs. In the valid condition, where the memory color contained the search target, the search RTs were faster both for the rDLPFC-TMS and the rPPC-TMS compared with the vertex-TMS session, reflecting enhanced WM guidance effects. A previous study demonstrated that patients with frontal damage show a stronger effect of the memorized stimulus on the visual search compared with healthy controls, particularly when the memorized cue contained the search target (Soto et al., 2006a). The authors proposed that frontal lobe structures are involved in separating relevant targets from irrelevant information, when both are held in memory. Similarly, other lesion studies have shown that prefrontal cortex appears to play a critical role in the ability to flexibly reallocate attention, depending on which stimulus is relevant for the task at hand (Rossi et al., 2007), and damage to this area can lead to difficulty in maintaining attention on task relevant information and in selectively inhibiting irrelevant stimuli (Stuss et al., 2001). Our findings suggest that a virtual lesion, induced by TMS stimulation, of the rDLPFC may disrupt the attentional control or separation of the WM cue from the search target, resulting in an enhanced WM guidance effect when the search target (‘T’) and the irrelevant memory cue are spatially overlapped (valid condition). Thus, subjects did not effectively distinguish between the task-relevant and task-irrelevant information (Erez and Duncan, 2015) after the disruption of the rDLPFC activity.

The rPPC is responsible for both the covert attention of WM contents and the overt attention of the search target (Rushworth et al., 2001), thus, stimulating rPPC may have disrupted the attention allocation induced by WM and the visual search. Furthermore, the rPPC has been suggested to suppress the distraction of irrelevant WM contents during a visual search task (Soto et al., 2012b). As hypothesized we showed that, compared with the vertex-TMS, TMS to the rPPC disrupted the suppression of irrelevant WM content, leading to faster search RTs in the valid condition. These findings extend those of an earlier study in which the left PPC was found to enhance the benefits of valid WM cues (Kiyonaga et al., 2014).

However, contrary to our hypothesis we did not observe increases of interference after stimulation of either the rDLPFC or the rPPC in the invalid condition. Previous studies have shown that a visual target elicits a stronger neural response in fronto-parietal regions when it matches, but not when it mismatches, the content of the WM (Soto et al., 2007; Gayet et al., 2017). Thus, the DLPFC and the PPC may be specifically involved in the processing of valid cued items, but not of targets in the invalid condition. TMS effects on behavior may depend on the initial activation state of the stimulated region (Silvanto et al., 2008; Sandrini et al., 2011; van de Ven and Sack, 2013). We propose that if the valid and invalid conditions are associated with distinct states in fronto-parietal regions, then the behavioral effect of fronto-parietal TMS may be dissociated across the two validity conditions, thus showing a lack of TMS interference in the invalid condition.

The TMS parameters (10 Hz, 5 pulses) used in the present study were expected to induce disruption effects. However, the search performance was enhanced after rDLPFC-TMS and rPPC-TMS in the valid condition, which is a counterintuitive behavioral result. One possibility is that the TMS disrupts processes that are normally detrimental to the experimental task (Tadin et al., 2011). Thus, rTMS may have disrupted the function of the rDLPFC in separating relevant search targets from irrelevant WM cue representations, and the function of the rPPC in suppressing distraction effects of irrelevant WM cues, both of which could have contributed to the observed enhanced WM effects, resulting in faster search RTs in the valid condition. In addition, we observed a trend for longer adjusted RTs after rDLPC-TMS and rPPC-TMS in the neutral condition. This is in line with evidence showing that stimulation of these sites disrupt the search task (Lane et al., 2011; Yan et al., 2016).

The memory test showed that accuracy was greater following the valid cued search compared to the neutral and invalid cued conditions. This pattern of results suggests that subjects may have used the reappearance of the WM cue to facilitate the subsequent memory recognition (Woodman and Luck, 2007). However, we did not observe any significant differences in accuracy between the neutral and invalid trials. These findings are in contrast to our initial hypothesis that the reappearance of a memory cue in the invalid trials during the retention interval would benefit the subsequent memory performance (Soto et al., 2012a). It is possible that the subjects’ attention did not shift toward the WM-match distractor and thus no cost effects were observed. In addition, no TMS effects were observed for the memory test, possibly since the rTMS was delivered during the search presentation rather than during the memory test presentation. Previous studies showed that the duration of the rTMS after-effects is short-lasting, about half the duration of the stimulation train (Robertson et al., 2003; Sandrini et al., 2011). In our study a duration of 500 ms (5 pulses of TMS delivered at 10 Hz), may not have affected the memory recognition.

Our results showed that stimulation of the rDLPFC and the rPPC induced a decrease of the search RTs in the valid condition, but showed no effects on the search performance in the neutral and invalid conditions. These findings suggest that these two cortical regions have an important function in biasing WM attentional capture, especially during valid trials, where the internal (item held in WM) and external signals (visual information in the search array) fully match the task goals (holding an item in WM and identifying the search target). Nevertheless, in the absence of direct neural evidence, and due to the relatively small number of trials in the experimental design, these interpretations remain speculative. However, our findings support the proposition that rDLPFC and rPPC are critical for controlling WM biases in human visual attention.

## Author Contributions

MW, PY, CW ZJ, JZ, and LL conceived and designed the experiments. MW and PY performed the experiments, analyzed the data, and wrote the main manuscript text. All authors reviewed the manuscript.

## Conflict of Interest Statement

The authors declare that the research was conducted in the absence of any commercial or financial relationships that could be construed as a potential conflict of interest.
